# A disproportionality analysis of FDA adverse event reporting system (FAERS) events for filgotinib

**DOI:** 10.1371/journal.pone.0338188

**Published:** 2025-12-15

**Authors:** Yinli Shi, Shuang Guan, Sicun Wang, Muzhi Li, Yanan Yu, Jun Liu, Weibin Yang, Zhong Wang

**Affiliations:** 1 Institute of Basic Research in Clinical Medicine, China Academy of Chinese Medical Sciences, Beijing, China; 2 Graduate School of China Academy of Chinese Medical Sciences, Beijing, China; Emory University School of Medicine, UNITED STATES OF AMERICA

## Abstract

**Background:**

Although filgotinib, a selective Janus kinase 1 inhibitor, has been increasingly applied in the treatment of inflammatory diseases, its comprehensive safety profile remains insufficiently characterized. Using data from the FAERS database covering Q1 2014 to Q2 2024, this study attempts to analyze adverse event signals linked to filgotinib and provide guidance for the safe and sensible clinical usage of filgotinib.

**Methods:**

From Q1 2014 to Q2 2024, information on adverse drug events (ADEs) associated with filgotinib was gathered. The reporting odds ratio (ROR), proportional reporting ratio (PRR), Bayesian confidence propagation neural network (BCPNN), and multi-item gamma Poisson shrinker (MGPS) were among the signal detection methods that were employed for analysis following data normalization.

**Results:**

Filgotinib was shown to be the main suspected medication in ADE reports, exposing 103 preferred terms (PTs) in 17 system organ classes (SOCs). Infections, gastrointestinal disorders, and musculoskeletal and connective tissue disorders were the most commonly reported adverse effects. Additionally, atrial fibrillation, alopecia, elevated serum creatinine, blood creatinine increased, pulmonary embolism, epididymitis, respiratory failure, and osteopenia were identified as potential disproportionate reporting signals for filgotinib, although these were not listed in the official drug label. Notable significant signals included large intestine erosion (ROR 2186.05, 95%CI_(ROR)_: 1015.94–4703.86, PRR 2176.18, 95%CI_(PRR)_: 1014.64–4667.42), mesenteric arterial occlusion (ROR 1832.17, 95%CI_(ROR)_: 897.68–3739.48, PRR 1822.71, 95%CI_(PRR)_: 896.17–3707.20), repetitive strain injury (ROR 1149.27, 95%CI_(ROR)_: 363.16–3637.01, PRR 1147.05, 95%CI_(PRR)_: 363.24–3622.15), oligoarthritis (ROR 755.02, 95%CI_(ROR)_: 310.74–1834.54, PRR 752.59, 95%CI_(PRR)_: 310.60–1823.51), and periostitis (ROR 676.03, 95%CI_(ROR)_: 319.36–1431.06, PRR 672.98, 95%CI_(PRR)_: 318.97–1419.87). The subgroup analysis identified obvious sex and age-specific trends in filgotinib-related adverse reactions, emphasizing a higher risk of renal disorders in females, a preponderance of gastrointestinal events in males, and age-dependent trends involving mesenteric occlusion, increased serum creatinine, and immunoglobulin reduction.

**Conclusion:**

While filgotinib demonstrates therapeutic efficacy, it is associated with a range of potential adverse events, underscoring the need for vigilant clinical monitoring. Particular attention should be given to gastrointestinal, cardiovascular, respiratory, and metabolic complications.

## 1. Introduction

Chronic inflammation in the joints and surrounding tissues is a hallmark of inflammatory arthritis (IA), a joint disease caused by a combination of environmental causes and hereditary predisposition [1]. Rheumatoid arthritis (RA), ankylosing spondylitis (AS), psoriatic arthritis (PsA), and various types of spondyloarthritis (SpA) are the main types of IA [2]. These diseases result from aberrant immune system activation, which triggers inflammatory signaling networks within cells. In addition to harming joints, this ongoing inflammatory reaction can also have an impact on other organs and bodily systems [3].

The Janus kinase-signal transducer and activator of transcription (JAK-STAT) pathway plays an essential role in cellular responses to external stimuli and is therefore considered central to various immune diseases [4,5]. Filgotinib is a second-generation JAK1-selective inhibitor that was created by Galapagos NV and Gilead Sciences [6]. In the European Union and Japan, filgotinib has been approved in a number of nations to treat inflammatory conditions [7]. When used to treat AS, filgotinib has some advantages over traditional JAK inhibitors, especially tofacitinib and upadacitinib [8,9]. These advantages include improved selectivity for JAK1, a more favorable safety profile, beneficial effectiveness, and a more flexible dosage schedule. One notable advantage of filgotinib is that it does not affect male fertility [10]. This makes it a particularly appealing therapy choice for people with AS who require long-term care or are concerned about their reproductive health. The TORTUGA trial demonstrated its efficacy and tolerability in patients with active AS [11]. Compared to other JAK inhibitors, filgotinib has shown a relatively favorable safety profile, which has also been supported by more recent evidence from clinical practice and observational research [12,13]. However, the possible adverse effects (AEs) linked to filgotinib in practical applications have garnered more attention as its use grows. Although the safety of filgotinib was assessed in clinical trials, certain uncommon or chronic adverse drug events (ADEs) are still poorly understood due to the rigorous inclusion criteria and small sample sizes in these investigations. Common AEs for filgotinib include nausea, dizziness, upper respiratory tract infections, and urinary tract infections, according to existing research [14–16]. Retrospective studies on JAK inhibitors indicate an increased risk of infection, subsequent safety trials have underscored the importance of early AE recognition and dose adjustments to reduce toxicity [17]. It is crucial to note that patient responses to filgotinib vary significantly due to individual health conditions, environmental factors, and genetic variants [18]. Despite the fact that filgotinib has shown positive effectiveness and a relatively low safety profile in several clinical studies, the information that is now available is mostly based on controlled research populations. Not enough is known about the real-world patterns of medication consumption and the range of adverse events linked to filgotinib. Specifically, there is currently a significant data gap regarding its long-term safety profile when compared to other JAK inhibitors.

The US Food and Drug Administration (FDA) Adverse Event Reporting System (FAERS) database provides support for the FDA post-marketing regulation of drugs and therapeutic biologics. It compiles information collected by the FDA on AEs and medication errors, enabling detailed analysis to identify potential risk factors, high-risk populations, and emerging safety issues. As a publicly accessible resource, FAERS gathers ADE reports submitted voluntarily by healthcare professionals, patients, and pharmaceutical manufacturers across various regions, offering a comprehensive reflection of ADE occurrences [19,20]. This study utilizes data mining techniques to analyze real-world adverse event signals associated with filgotinib, providing valuable insights and guidance for its clinical use.

## 2. Materials and methods

### 2.1. Data source

Data on post-market adverse drug reactions is compiled by the FAERS database, which has been openly available since 2004 and is updated every three months. Comprising patient demographic and administrative details (DEMO), housing drug-specific information (DRUG), coded representations of reported adverse events (REAC), reflecting patient outcomes (OUTC), indicating sources of reports (RPSR), documenting therapy initiation and cessation dates for reported drugs (THER), and outlining indications for drug administration (INDI) are the seven separate files that make up the FAERS dataset. “Jyseleca” and “Filgotinib” were used as keywords in a thorough search to find all related adverse event reports from Q1 2014 to Q2 2024. The signal analysis for this study encompassed data from the initial post-marketing quarters after filgotinib’s formal regulatory approval. The international council for harmonization of technical requirements for pharmaceuticals for Medical Dictionary for Regulatory Activities (MedDRA), version 24.0, was consulted in order to classify and analyze these reports according to the preferred term (PT) and the system organ class (SOC).

As the analysis involved only de-identified, retrospective data, no ethics committee approval or informed consent was required, in accordance with the International Council for Harmonisation Good Clinical Practice (ICH-GCP) guidelines and relevant regulatory requirements.

### 2.2. Data preprocess

The reports that were retrieved suggested that filgotinib was the primary suspect associated with ADE. These reports include a wide range of information, such as the patient’s age and sex, the date of presentation, records of prescription usage, treatment outcomes, reporting sources, and more. For cases with duplicate records, a CASEID/ISR deduplication strategy was applied: when multiple ISRs corresponded to the same CASEID, only the most recent ISR record was retained, and all other duplicates were removed to minimize potential bias in ADE signal detection. Name mapping and normalization of filgotinib was carried out utilizing the Medex_UIMA_1.8.3 system in conjunction with an unique drug terminology lexicon to guarantee consistency in data standards; the synonyms “Filgotinib,” “Jyseleca,” “GLPG0634,” and “GS-6034” were kept.

### 2.3. Data signal filtering and classification

As detailed in S1 Table, nine non-validated signals were then eliminated, including events associated with primary conditions (such as moderate-to-severe rheumatoid arthritis and ulcerative colitis), non-relevant adverse drug reactions (such as affordability issues, insurance limitations, non-treatment-related reactions), and drug or device misuse (such as product name confusion, intentional misuse). PT with a report count of ≥3 was chosen for the study’s initial screening. Signals were coded, classified, and localized using MedDRA’s PT and SOC in order to identify the specific SOC implicated in the adverse event signal.

### 2.4. Statistical analysis

The reporting odds ratio (ROR), the medicines healthcare products regulatory agency (MHRA), the bayesian confidence propagation neural network (BCPNN), and the multi-item gamma Poisson shrinker (MGPS) method were the four methods utilized for ADE signal mining [21–23]. ROR, which is commonly used because of its ease of use and high sensitivity, was deemed significant when the lower bound of the 95% 9 CI exceeded 1. The MHRA method, which is an extension of the proportional reporting ratio (PRR), was regarded as positive when PRR > 2, chi-square > 4, and the number of reports ≥ 3. MGPS utilized the empirical Bayes geometric mean (EBGM) with a threshold of EBGM05 > 2, whereas the BCPNN approach applied the information component (IC) to identify signals. A positive signal was defined as IC025 > 0. An AE was deemed a positive drug-event signal in this study if it satisfied the threshold of at least one of the four signal detection algorithms; if all four algorithms satisfied the predetermined criteria, the AE was categorized as having a strong correlation, which reduced the possibility of false-positive signals. 2 × 2 contingency tables were used to determine the parameters for the ROR and associated computations.

**Fig 1:**
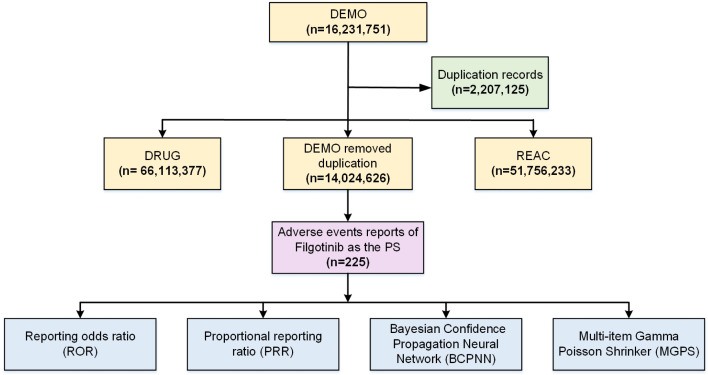
Flow chart for filgotinib ADE identification from the FAERS database.

Fig 1 illustrates a comprehensive flowchart detailing the process of data extraction for ADEs associated with filgotinib from the FAERS database. The time-to-onset (TTO) analysis included only reports with clearly documented drug start and end dates as well as event onset dates. To enhance the accuracy of the findings, subgroup analyses were performed stratified by sex and age. Sensitivity analyses were restricted to cases reported by healthcare professionals to minimize potential confounding bias. Reports with missing values for age, sex, or event onset date were included in the overall disproportionality analysis but excluded from variable-specific subgroup or temporal trend analyses, thereby reducing the impact of missing information on the results. Tables 1 and 2 provide the formulas for the four methods as well as the criteria for detecting signals. R Studio (version 4.3.1) and Microsoft Excel 2023 were utilized for statistical analyses and data visualizations.

**Table 1:** Algorithms based on four grid table.

	Drug-related ADEs	Non-drug-related ADEs	Total
Drug	a	b	a + b
Non-drug	c	d	c + d
Total	a + c	b + d	N = a + b + c + d

a, number of reports containing both the target drug and target adverse drug reaction; b, number of reports containing other adverse drug reaction of the target drug; c, number of reports containing the target adverse drug reaction of other drugs; d, number of reports containing other drugs and other adverse drug reactions.

**Table 2:** Four major algorithms used for signal detection.

Algorithms	Equation	Criteria
ROR	ROR = ad/bc	lower limit of 95% CI > 1, N > 3
	SE (ln ROR)=(1/a + 1/b + 1/c + 1/d)^0.5	
	95%CI = e^ln(ROR)±1.96(1/a + 1/b + 1/c + 1/d)^0.5^	
PRR	PRR = a(c + d)/c/(a + b)	PRR > 2, χ^2 > ^4, N > 3
	χ^2^=[(ad-bc)^2](a + b + c + d)/[(a + b)(c + d)(a + c)(b + d)]	
BCPNN	IC = log_2_[a(a + b + c + d)/(a + c)(a + b)]	IC025 > 0
	E(IC)=log_2_[(a + γij)(a + b + c + d + α)(a + b + c + d + β)]/[(a + b + c + d + γ)(a + b + αi)(a + c + βj)]	
	γ = γij(a + b + c + d + α)(a + b + c + d + β)	
	95%CI = E(IC) ± 2V(IC)^0.5	
MGPS	EBGM = a(a + b + c + d)/(a + c)/(a + b)	EBGM05 > 2
	95%CI = e^ln(EBGM)±1.96(1/a + 1/b + 1/c + 1/d)^0.5^	

95%CI, 95% confidence interval; N, the number of reports; χ2, chi-squared; IC, information component; IC025, the lower limit of 95% CI of the IC; E(IC), the IC expectations; EBGM, empirical Bayesian geometric mean; EBGM05, the lower limit of 95% CI of EBGM.

## 3. Results

### 3.1. Descriptive analysis

From the second quarter of 2014 to the second quarter of 2024, a total of 16,231,751 adverse event reports were retrieved from the FAERS database. After removing 2,207,125 duplicate entries, 14,024,626 unique and valid reports were retained for analysis. Among these, 225 reports identified filgotinib as the primary suspect drug, corresponding to 870 ADEs. The data screening and cleaning workflow is illustrated in Fig 1. Between 2014 and 2016, there were no reports of filgotinib-related AEs. Nevertheless, between 2017 and 2021, the number of AE reports involving filgotinib increased annually. The years 2022–2024 saw a decline in this pattern. Interestingly, a far greater percentage of filgotinib adverse events (64.89% vs. 31.11%) were reported by female patients than by male patients. A thorough examination of age-related AE trends was limited since only 51.56% of reports contained precise age information. Nonetheless, 33.33% of reports with explicit age data came from people between the ages of 18 and 65. The majority of reports (59.56%) were submitted by physician, with health professional coming in second (24.44%). Germany accounted for the highest proportion of reports (51.56%), followed by the Kingdom of the Netherlands (9.3%), Japan (8.89%), Canada (5.33%), and Poland (4.89%). In terms of clinical outcomes, the most frequently reported adverse events—excluding those with unidentified significance—resulted in hospitalization (initial or prolonged) in 82.22% of cases, and death in 6.22% of cases. Further information can be found in Table 3.

**Table 3:** Basic information on ADE reports related to filgotinib.

Category	Count	Proportion
***Patient outcome***		
Hospitalization Initial or Prolonged	185	82.22%
Other serious (Important Medical Event)	21	9.33%
Death	14	6.22%
Disability	5	2.22%
***Patient occupation***		
Physician	134	59.56%
health professional	55	24.44%
Consumer	28	12.44%
Other health-professional	6	2.67%
Unknown	2	0.89%
***Reporter country (Top five)***		
Germany	116	51.56%
The Kingdom of the Netherlands	21	9.33%
Japan	20	8.89%
Canada	12	5.33%
The Republic of Poland	11	4.89%
***Sex***		
Male	70	31.11%
Female	146	64.89%
Unknown	9	4.00%
***Age***		
<18	1	0.44%
18-65	75	33.33%
>65	40	17.78%
Unknown	109	48.44%
***Adverse event year***		
2017	10	4.44%
2018	14	6.22%
2019	14	6.22%
2020	21	9.33%
2021	55	24.44%
2022	29	12.89%
2023	35	15.56%
2024	1	0.44%
Unknown	46	20.44%

Filgotinib-related adverse event reports increased after the drug’s debut on the market and then gradually stabilized during the course of the post-marketing surveillance period. However, the general trend points to a possible Weber effect in the initial post-marketing stage.

### 3.2. Signal ADE mining

To ensure the robustness of the findings, we required consistency across all four predefined algorithms and excluded invalid signals. Based on this stringent criterion, a total of 96 risk signals were ultimately identified. It should be noted that for PTs with a limited number of reports, the corresponding ROR values may be subject to inflation effects. Therefore, in interpreting the results, greater emphasis was placed on the more stable IC and EBGM metrics, in order to minimize the impact of statistical instability on the conclusions.

### 3.3. SOCs of ADE signals and frequency analysis

Four algorithms were used in order to identify adverse event signals based on the predetermined screening thresholds. Table 4 indicates that a total of 870 adverse reaction reports involving 17 SOCs were found in relation to this medication. Infections and infestations (142 reports, 14 signals), musculoskeletal and connective tissue disorders (110 reports, 11 signals), gastrointestinal disorders (92 reports, 15 signals), respiratory, thoracic and mediastinal disorders (84 reports, 7 signals), and investigations (236 reports, 19 signals) were the SOCs with the highest report counts. This distribution roughly matches ADE signal strength-based ranks. With a primary focus on musculoskeletal and connective tissue diseases and gastrointestinal disorders, the screening results were highly uniform across the four algorithms. Notably, several adverse events exhibited high signal intensity despite the limited number of case reports. These included large intestine erosion (n = 7, ROR 2186.05, PRR 2176.18, IC 11.00, EBGM 2042.62), mesenteric arterial occlusion (n = 8, ROR 1832.17, PRR 1822.71, IC 10.75, EBGM 1728.08), repetitive strain injury (n = 3, ROR 1149.27, PRR 1147.05, IC 10.11, EBGM 1108.85), oligoarthritis (n = 5, ROR 755.02, PRR 752.59, IC 9.52, EBGM 735.96), and periostitis (n = 7, ROR 676.03, PRR 672.98, IC 9.37, EBGM 659.65) (Table 5).

**Table 4:** System organ class (SOC) distribution: the number of ADE reports and the number of signals for filgotinib.

SOC	ADE signals (%)	ADE reports (%)
Blood and lymphatic system disorders	2 (2.08)	14 (1.60)
Cardiac disorders	1 (1.04)	24 (2.76)
Endocrine disorders	1 (1.04)	5 (0.57)
Gastrointestinal disorders	15 (15.63)	92 (10.57)
General disorders and administration site conditions	6 (6.25)	49 (5.63)
Infections and infestations	14 (14.58)	142 (16.32)
Injury, poisoning and procedural complications	4 (4.17)	13 (1.49)
Investigations	19 (19.79)	236 (27.13)
Metabolism and nutrition disorders	3 (3.13)	24 (2.76)
Musculoskeletal and connective tissue disorders	11 (11.46)	110 (12.64)
Neoplasms benign, malignant and unspecified (incl cysts and polyps)	3 (3.13)	12 (1.38)
Nervous system disorders	2 (2.08)	17 (1.95)
Respiratory, thoracic and mediastinal disorders	7 (7.29)	84 (9.66)
Skin and subcutaneous tissue disorders	3 (3.13)	28 (3.22)
Surgical and medical procedures	2 (2.08)	9 (1.03)
Vascular disorders	3 (3.13)	11 (1.26)
**Total**	96	870

**Table 5:** PT signal detection results of top 20 ADEs based on signal strength about filgotinib.

PTs	n	a	b	c	d	PRR	X^2^	IC	IC025	EBGM	EBGM05	ROR	LowerCI	UpperCI
Rhesus antigen positive^#^	14(1.6%)	14	1535	1	51526622	465702.209	434653.655	14.922	3.154	31047.747	4080.081	469949.647	61757.541	3576124.718
Blood albumin increased^#^	14(1.6%)	14	1535	629	51525994	740.385	10112.460	9.500	3.127	724.286	425.472	747.129	438.891	1271.846
Periostitis^#^	7(0.8%)	7	1542	346	51526277	672.980	4603.763	9.366	1.959	659.655	311.621	676.031	319.357	1431.056
Intestinal anastomosis complication^#^^,^^△^	4(0.5%)	4	1545	208	51526415	639.701	2502.691	9.294	1.018	627.650	233.072	641.354	238.161	1727.130
Medullary thyroid cancer^#^	5(0.6%)	5	1544	261	51526362	637.250	3116.569	9.288	1.391	625.290	257.731	639.310	263.510	1551.053
Ileectomy^#^	5(0.6%)	5	1544	265	51526358	627.631	3070.250	9.267	1.391	616.027	253.945	629.660	259.565	1527.449
Blood immunoglobulin M decreased^#^	14(1.6%)	14	1535	965	51525658	482.593	6632.239	8.894	3.113	475.706	280.007	486.985	286.646	827.342
Epididymitis^#^	14(1.6%)	14	1535	1002	51525621	464.773	6389.698	8.840	3.111	458.382	269.848	469.003	276.100	796.681
Immunoglobulins decreased^#^	13(1.5%)	13	1536	1372	51525251	315.188	4033.376	8.287	2.970	312.239	180.424	317.847	183.664	550.062
Chronic respiratory failure^#^	8(0.9%)	8	1541	861	51525762	309.077	2434.069	8.259	2.165	306.241	152.385	310.677	154.592	624.354
Gastrointestinal erosion^#^^,^^△^	4(0.5%)	4	1545	458	51526165	290.519	1144.109	8.170	1.007	288.012	107.498	291.269	108.713	780.378
Ileal ulcer^#^	7(0.8%)	7	1542	812	51525811	286.762	1976.356	8.151	1.939	284.320	134.886	288.060	136.660	607.187
Bronchitis bacterial^#^	7(0.8%)	7	1542	849	51525774	274.265	1890.327	8.088	1.938	272.030	129.074	275.506	130.723	580.645
Terminal ileitis^#^^,^^△^	4(0.5%)	4	1545	487	51526136	273.219	1076.064	8.082	1.006	271.002	101.174	273.924	102.265	733.722
Mean cell haemoglobin increased^#^	14(1.6%)	14	1535	1762	51524861	264.303	3643.475	8.035	3.080	262.228	154.614	266.705	157.253	452.336

# : It indicates that AE all meet the standard thresholds of the four algorithms and have a strong correlation.

△ : This suggests that the number of reported cases is fewer than five.

According to the manufacturer’s guidelines, the following severe and frequently reported ADEs had high signal intensity: dyslipidaemia (n = 14, ROR 109.89, 95% CI: 64.87–186.16), urinary tract infection (n = 20, ROR 4.36, 95% CI: 2.80–6.77), herpes zoster (n = 13, ROR 7.98, 95% CI: 4.62–13.78), pneumonia (n = 30, ROR 5.24, 95% CI: 3.65–7.52), and white blood cell count decreased (n = 14, ROR 4.70, 95% CI: 2.78–7.95). Significantly, as shown in Table 6, possible novel ADE signals that were not covered in the package insert were found, such as atrial fibrillation (n = 24, ROR 10.52, 95% CI: 7.03–15.75), alopecia (n = 21, ROR 3.58, 95% CI: 2.33–5.51), blood creatinine increased (n = 17, ROR 11.26, 95% CI: 6.98–18.16), pulmonary embolism (n = 17, ROR 8.96, 95% CI: 5.56–14.46), epididymitis (n = 14, ROR 469.00, 95% CI: 276.10–796.68), acute respiratory failure (n = 14, ROR 31.29, 95% CI: 18.48–52.97), and osteopenia (n = 14, ROR 27.63, 95% CI: 16.32–46.78).

**Table 6:** PT signal detection results of the top 20 ADEs based on the number of reports about filgotinib.

PTs	n	a	b	c	d	PRR	X^2^	IC	IC025	EBGM	EBGM05	ROR	LowerCI	UpperCI
COVID-19^#^	30(3.4%)	30	1519	193527	51333096	5.16	100.88	2.37	1.65	5.16	3.59	5.24	3.65	7.52
Atrial fibrillation^#^	24(2.8%)	24	1525	76938	51449685	10.38	203.59	3.37	2.33	10.37	6.93	10.52	7.03	15.75
Alopecia^#^	21(2.4%)	21	1528	197027	51329596	3.55	38.52	1.83	1.04	3.55	2.30	3.58	2.33	5.51
Urinary tract infection^#^	20(2.3%)	20	1529	154286	51372337	4.31	51.03	2.11	1.26	4.31	2.77	4.36	2.80	6.77
Osteoarthritis^#^	19(2.2%)	19	1530	38766	51487857	16.30	273.01	4.03	2.55	16.30	10.36	16.49	10.49	25.93
Interstitial lung disease^#^	18(2.1%)	18	1531	39224	51487399	15.27	240.02	3.93	2.45	15.26	9.59	15.43	9.70	24.56
Blood creatinine increased^#^	17(2.0%)	17	1532	50746	51475877	11.14	157.07	3.48	2.14	11.14	6.91	11.26	6.98	18.16
Glomerular filtration rate decreased^#^	17(2.0%)	17	1532	10476	51516147	53.98	882.72	5.75	3.09	53.89	33.40	54.57	33.82	88.04
Pulmonary embolism^#^	17(2.0%)	17	1532	63701	51462922	8.88	118.95	3.15	1.94	8.88	5.50	8.96	5.56	14.46
Haematocrit decreased^#^	15(1.7%)	15	1534	15097	51511526	33.05	465.89	5.05	2.73	33.02	19.85	33.36	20.06	55.49
Blood albumin increased^#^	14(1.6%)	14	1535	629	51525994	740.39	10112.46	9.50	3.13	724.29	425.47	747.13	438.89	1271.85
Blood immunoglobulin G decreased^#^	14(1.6%)	14	1535	2396	51524227	194.37	2677.68	7.59	3.05	193.24	114.00	196.13	115.70	332.46
Blood immunoglobulin M decreased^#^	14(1.6%)	14	1535	965	51525658	482.59	6632.24	8.89	3.11	475.71	280.01	486.99	286.65	827.34
Blood potassium increased^#^	14(1.6%)	14	1535	12439	51514184	37.44	496.08	5.22	2.70	37.40	22.09	37.77	22.31	63.95
Dyslipidaemia^#^	14(1.6%)	14	1535	4276	51522347	108.91	1492.12	6.76	2.98	108.56	64.09	109.89	64.87	186.16
Epididymitis^#^	14(1.6%)	14	1535	1002	51525621	464.77	6389.70	8.84	3.11	458.38	269.85	469.00	276.10	796.68
Mean cell haemoglobin increased^#^	14(1.6%)	14	1535	1762	51524861	264.30	3643.47	8.03	3.08	262.23	154.61	266.70	157.25	452.34
Protein urine present^#^	14(1.6%)	14	1535	4720	51521903	98.67	1349.58	6.62	2.96	98.38	58.08	99.56	58.78	168.63
Red cell distribution width increased^#^	14(1.6%)	14	1535	4653	51521970	100.09	1369.35	6.64	2.97	99.79	58.91	100.99	59.62	171.06
Rhesus antigen positive^#^	14(1.6%)	14	1535	1	51526622	465702.21	434653.66	14.92	3.15	31047.75	4080.08	469949.65	61757.54	3576124.72
Vitamin D decreased^#^	14(1.6%)	14	1535	8367	51518256	55.66	750.35	5.80	2.83	55.57	32.82	56.16	33.17	95.09

# : It indicates that AE all meet the standard thresholds of the four algorithms and have a strong correlation.

### 3.4. PTs associated with gastrointestinal disorders

**Fig 2:**
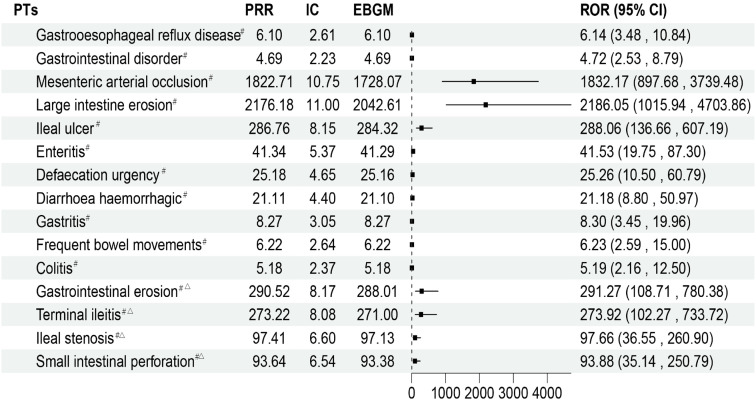
The status of PTs related to gastrointestinal disorders. ^#^: It indicates that AE all meet the standard thresholds of the four algorithms and have a strong correlation. ^△^:This suggests that the number of reported cases is fewer than five.

Fig 2 displays the PT linked to gastrointestinal disorders. Despite the small number of case reports, we discovered new ades added to FDA approved drug labels, such as mesenteric arterial occlusion (n = 8, ROR 1832.17, 95%CI: 897.68–3739.48) and large intestine erosion (n = 7, ROR 2186.05, 95%CI: 1015.94–4703.86), which displayed exceptionally high signal intensity. Other possible novel ADE risk indicators include gastrointestinal erosion, ileal ulcer, terminal ileitis, ileal stenosis, and small intestinal perforation.

### 3.5. PTs associated with respiratory, thoracic, and mediastinal disorders

**Fig 3:**
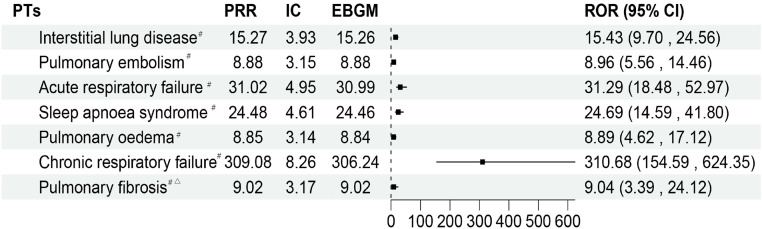
The status of PTs related to respiratory, thoracic, and mediastinal disease. ^#^: It indicates that AE all meet the standard thresholds of the four algorithms and have a strong correlation. ^△^:This suggests that the number of reported cases is fewer than five.

The PTs for respiratory, thoracic, and mediastinal disorders are displayed in Fig 3. According to the findings, the maximum signal strength was seen in chronic respiratory failure (n = 8, ROR 310.68, 95% CI: 154.59–624.35). Furthermore, there was a high frequency of reports and significant signal intensity for acute respiratory failure (n = 14, ROR 31.29, 95% CI: 18.48–52.97), sleep apnea syndrome (n = 14, ROR 24.69, 95% CI: 14.59–41.80), interstitial lung disease (n = 18, ROR 15.43, 95% CI: 9.70–24.56), and pulmonary embolism (n = 17, ROR 8.96, 95% CI: 5.56–14.46), which may indicate new ADE risk signals.

### 3.6. PTs associated with infections and infestations

**Fig 4:**
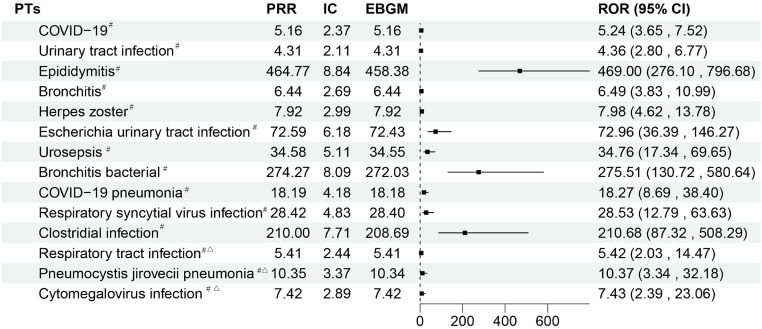
The status of PTs related to infections and infestations. ^#^: It indicates that AE all meet the standard thresholds of the four algorithms and have a strong correlation. ^△^:This suggests that the number of reported cases is fewer than five.

Several novel ADE risk signals for filgotinib were discovered on the infections and infestations. Clostridial infection (n = 5, ROR 210.68, 95%CI: 87.32–508.29), bronchitis bacterial (n = 7, ROR 275.51, 95%CI: 130.72–580.64), and epididymitis (n = 14, ROR 469.00, 95%CI: 276.10–796.68) were shown to have high risk signal intensity. As a result of the COVID-19 pandemic, bronchitis, herpes zoster, urosepsis, urinary tract infection, and cytomegalovirus infection are all regarded to be potential novel ADE signs (Fig 4).

### 3.7. PTs associated with musculoskeletal and connective tissue disorders

**Fig 5:**
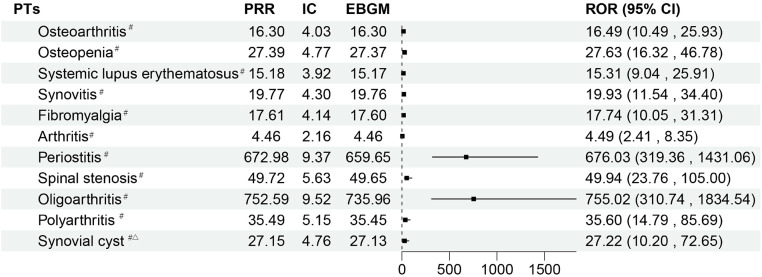
The status of PTs related to musculoskeletal and connective tissue disorders. ^#^: It indicates that AE all meet the standard thresholds of the four algorithms and have a strong correlation. ^△^:This suggests that the number of reported cases is fewer than five.

According to the data, which are displayed in Fig 5, there have been case reports with increased rates of fibromyalgia, osteoarthritis, systemic lupus erythematosus, osteopenia, and synovitis. Although there were just a few case reports for oligoarthritis (n = 5, ROR 755.02, 95%CI: 310.74–1834.54) and periostitis (n = 7, ROR 676.03, 95%CI: 319.36–1431.06) in the overall diseases category, it was discovered that there was a very significant risk signal.

### 3.8. Time to onset (TTO) analysis

**Fig 6:**
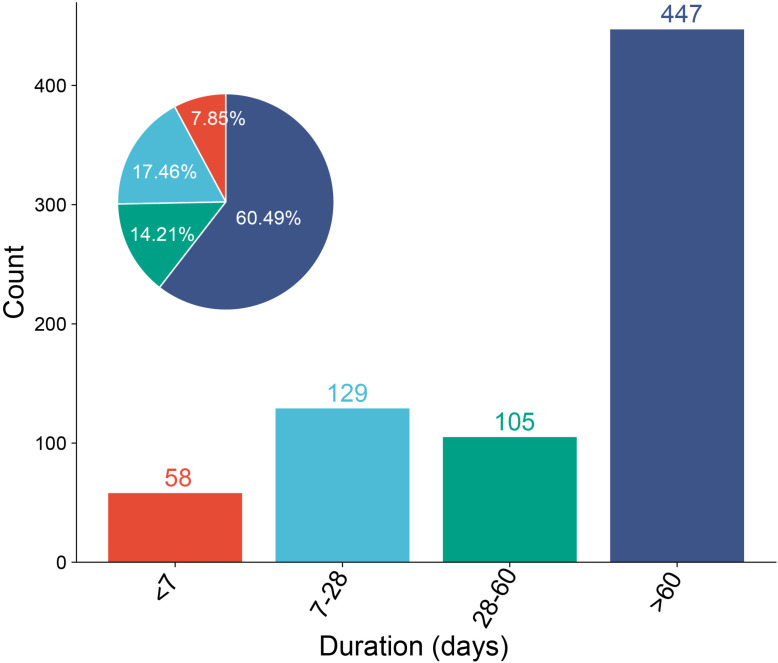
Time to onset of AEs associated with filgotinib.

After excluding false positives, data from the database identified a total of 739 cases reporting the onset time of filgotinib-related AEs. Of the cases with available onset time data, 447 cases (60.49%) occurred more than two months after initiating filgotinib treatment. An additional 129 cases (17.46%) were reported within 1–4 weeks of treatment initiation, while 105 cases (14.21%) emerged between 1 and 2 months. Notably, 58 ADEs, accounting for 7.85% of all cases, occurred within the first week of treatment (Fig 6).

### 3.9. Subgroup analysis

**Fig 7:**
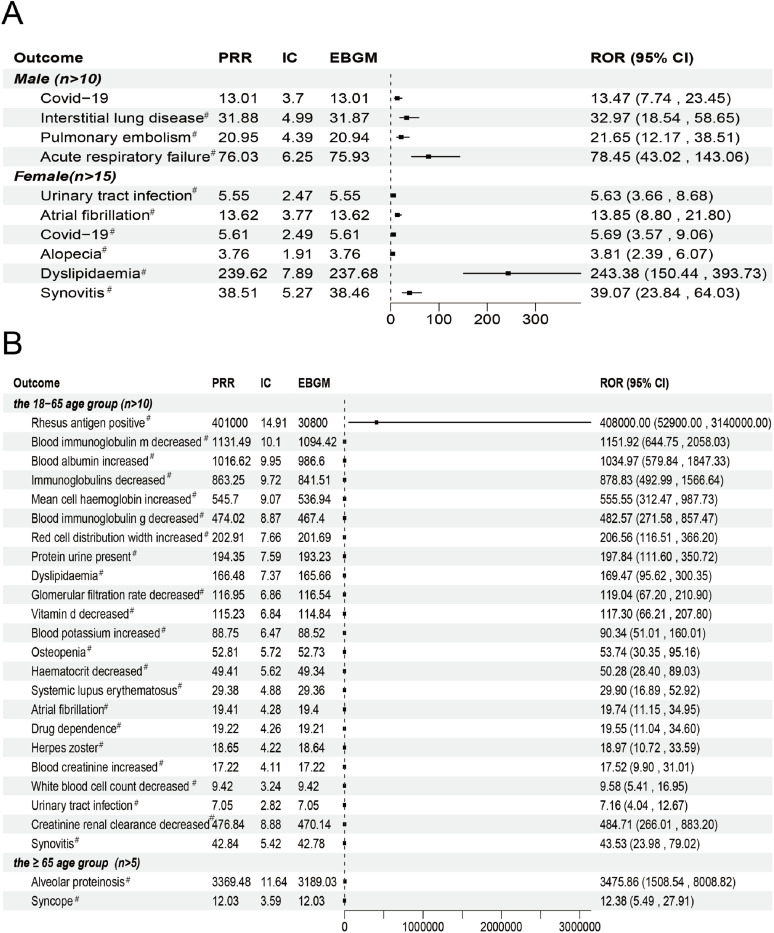
Gender and age differences in filgotinib safety. (A) Gender‐based subgroup analysis and (B) age‐based subgroup analysis were conducted according to the number of reports. ^#^: It indicates that AE all meet the standard thresholds of the four algorithms and have a strong correlation.

The ROR algorithm was used independently to AEs linked to filgotinib in male and female patients in order to investigate sex-specific variations in the drug’s safety signals. Males reported 155 adverse events (S2 Table), whereas females noticed 573 AEs (S3 Table). This result suggests that the observed differences may reflect underlying sample distribution patterns rather than a definitive effect modification. In addition to a number of sex-specific signals, a comparison of AEs by sex showed 45 overlapping events that were seen in both the male and female subgroups. Large intestine erosion (n = 6, ROR 4404.92, 95%CI: 1863.74–10411.00, *P* < 0.001), mesenteric arterial occlusion (n = 5, ROR 2404.85, 95%CI: 964.45–5996.49), oligoarthritis (n = 4, ROR 1699.26, 95%CI: 619.06–4664.34), blood albumin increased (n = 12, ROR 917.99, 95%CI: 515.21–1635.64) and blood immunoglobulin m decreased (n = 12, ROR 839.67, 95%CI: 471.60–1495.01) were the top five AEs that were unique to females. On the other hand, acute respiratory failure (n = 11, ROR 12683.40, 95%CI: 3598.47–44704.40), blood cholesterol increased (n = 3, ROR 1880.19, 95%CI: 766.33–4613.02), bronchitis bacterial (n = 4, ROR 1001.40, 95%CI: 410.95–2440.19), chronic respiratory failure (n = 6, ROR 1002.16, 95%CI: 370.73–2709.02) and crohn’s disease (n = 5, ROR 163.34, 95%CI: 67.48–395.41) were the top five AEs that were specific to males. Among the common adverse events, men were more frequently affected by frequent bowel movements, gastrointestinal disorders, interstitial lung disease, and pulmonary embolism. In contrast, females were more likely to present with decreased glomerular filtration rate, increased blood creatinine, osteoarthritis, gastritis, and gastroesophageal reflux disease (*P* < 0.001, Fig 7A). Nevertheless, given the high prevalence of comorbidities in patients with rheumatoid arthritis and related disorders, the observed subgroup trends might partly reflect underlying health conditions rather than drug-specific effects.

Additionally, we evaluated age-related variations in the safety signals associated with filgotinib, concentrating on the adult (18–65 years) and senior (≥65 years) populations. 363 AEs were found in the 18–65 age group (S4 Table), whereas 50 AEs were found in the ≥ 65 age group (S5 Table). Among 18–65 age group, immunoglobulins decreased (n = 12, ROR 31.19, 95%CI: 13.96–69.71), blood albumin increased (n = 12, ROR 1034.97, 95%CI: 579.84–1847.33), herpes zoster (n = 12, ROR 66.78, 95%CI: 24.97–178.63), urinary tract infection (n = 12, ROR 8.31, 95%CI: 2.67–25.83) and protein urine present (n = 12, ROR 23.05, 95%CI: 8.62–61.62) were the most often reported adverse events. Alveolar proteinosis (n = 6, ROR 5762.79, 95%CI: 2278.61–14574.60), syncope (n = 6, ROR 15.22, 95%CI: 4.87–47.61), escherichia urinary tract infection (n = 5, ROR 12.38, 95%CI: 5.49–27.91), dehydration (n = 5, ROR 49.72, 95%CI: 20.45–120.86), mesenteric arterial occlusion (n = 5, ROR 61.17, 95%CI: 19.55–191.45), respiratory syncytial virus infection (n = 5, ROR 29.05, 95%CI: 9.28–90.89) and acute kidney injury (n = 5, ROR 3475.86, 95%CI: 1508.54–8008.82) were among the most frequently reported AEs in the ≥ 65 age group (Fig 7B).

### 3.10. Sensitivity analysis

**Fig 8:**
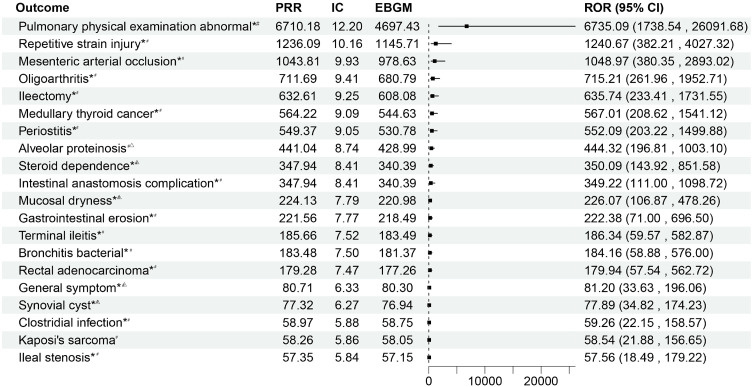
Sensitivity analysis based on ADRs reported by physicians. ^#^: It indicates that AE all meet the standard thresholds of the four algorithms and have a strong correlation. *: The PTs suggest that the sensitivity analysis overlaps with the previously mentioned disproportionate analysis. ^△^:This suggests that the number of reported cases is fewer than five.

A sensitivity analysis was carried out by limiting the data sources in order to reduce the possibility of influence from differences in report quality and false-positive signals. By choosing adverse event reports that were only filed by physicians, we were able to find 750 individual case reports with 275 AEs in total. The distributions of age, sex, and the percentage of severe incidents did not change significantly, according to baseline characteristic analysis (S6 Table). Pulmonary physical examination abnormal (n = 3, ROR 6735.09, 95%CI: 1738.54–26091.70), repetitive strain injury (n = 3, ROR 1240.67, 95%CI: 382.21–4037.32), mesenteric arterial occlusion (n = 4, ROR 1048.97, 95%CI: 380.35–2893.02), oligoarthritis (n = 4, ROR 715.21, 95%CI: 261.96–1952.71) and medullary thyroid cancer (n = 4, ROR 567.01, 95%CI: 208.62–1541.12), and gastrointestinal, respiratory, musculoskeletal, and connective tissue problems were among the persistently documented adverse events (Fig 8). A number of consistent signals, such as atrial fibrillation, bronchitis, gastrooesophageal reflux disease, glomerular filtration rate decreased and mesenteric arterial occlusion, were seen when compared to PTs mentioned in Tables 5 and 6. In S7 Table, these consistently found PTs were indicated by asterisks, signifying higher statistical robustness. A total of 157 adverse event reports were found after possible false-positive instances were eliminated. In accordance with the results from the earlier analysis, the majority of these instances (n = 100, 58.82%) happened in the first two months after filgotinib treatment was started.

## 4. Discussion

In clinical practice, non-steroidal anti-inflammatory medicines (NSAIDs) are frequently replaced with filgotinib, a biologic disease-modifying antirheumatic medication [24,25]. Drug-drug interactions are less likely when filgotinib is metabolized by carboxylesterase isoform 2, which produces metabolites that have no reliance on the hepatic enzyme CYP450 [26]. When compared to other JAK inhibitors, filgotinib shows unique safety features. Real-world studies have linked first- and third-generation JAK inhibitors, represented by tofacitinib and upadacitinib, respectively, to an increased risk of infections, cardiovascular events, and thrombotic problems. On the other hand, because of its greater selectivity for JAK1, filgotinib may give a more favorable safety profile by partially reducing JAK2-mediated hematologic toxicities and JAK3-related immunosuppressive effects. This study used retrospective post-marketing data from the FAERS database to do a systematic analysis of AE warnings related to filgotinib, a selective JAK1 inhibitor. The findings enhance knowledge for clinical application by offering comprehensive insights into the kind and frequency of filgotinib-related adverse events.

According to this study, female patients experienced filgotinib-related AEs more frequently than male patients did (146 cases, 64.89%, vs. 70 cases, 31.11%). Female individuals with RA, AS, and UC typically exhibit a lower healing rate than male patients, despite variations in the gender distribution of these patients [27–30]. According to Martinez-Molina et al.‘s multivariate Cox analysis, female gender may be linked to higher prognostic risk and more treatment interruptions in RA patients [31]. Although there are not many research on filgotinib-related AEs in female patients, patient gender should be taken into account when assessing the drug’s risk-benefit profile [32,33]. The analysis also showed that AEs were more frequent in adults aged 18–65. Epidemiological data suggest that RA, AS, and UC typically present between ages 20 and 55 [34–36]. Factors like frequent comorbidities, unhealthy lifestyle habits, emotional stress, and environmental influences may contribute to an increased AE risk in this age group. With societal aging, RA and UC incidence among the elderly is also rising, contributing to a greater number of AE reports [37]. A pharmacokinetic study by Namour et al. found that in filgotinib-treated patients over 75, exposure to the major metabolite (AUCτ) was 1.3 times higher than in patients aged 40–50 receiving the same dose, potentially explaining an increased incidence of serious infections [38]. This aligns with findings in those over 65, who accounted for 17.78% of cases in this study’s descriptive analysis. Since filgotinib’s original clearance in the European Union and Japan, AE reports have mostly focused on Europe and Asia. The drug’s indications were consistent across geographies, with no notable off-label use documented. Filgotinib was connected with significant outcomes such as hospitalization, life-threatening events, disability, and death. The relatively low incidence of life-threatening AEs is consistent with earlier safety findings, most likely due to filgotinib’s excellent selectivity for JAK1, which reduces the adverse effects associated with JAK2 inhibition [39,40]. The majority of filgotinib-related AEs happened during the first two months of treatment, according to time-to-event analysis. This emphasizes the necessity of careful patient monitoring and prompt dose modifications during this time to reduce side effects.

This study used the FAERS database to identify 870 reports that could be associated with filgotinib across 17 SOCs, including digestive, infectious, musculoskeletal, respiratory, cardiac, and skin disorders, as well as general diseases and administration-site reactions. This research found risk signals for rheumatic and digestive disorders such as oligoarthritis, periostitis, mesenteric arterial occlusion, large intestine erosion, gastrointestinal erosion, and ileal ulcer. These signs were lacking from filgotinib’s mentioned indications and recognized side effects, raising concerns about potential disproportionate reporting signals involving rheumatic immunological and gastrointestinal events in association with filgotinib use. While there have been few reports of long-term filgotinib treatment aggravating inflammatory joint pain and digestive disorders, the detected signals merit further investigation. The findings point to a complicated mechanism by which filgotinib modulates immune responses across numerous organ systems, potentially associated with increased reporting of gastrointestinal events and peripheral joint inflammation [41,42]. Although additional research is needed to completely understand these hazards, these findings highlight the necessity of raising clinician awareness and monitoring filgotinib’s impact on immune-mediated diseases.

The data point to risk factors for filgotinib-associated illnesses such as herpes zoster, bronchial infections, urinary tract infections, and pneumonia. These adverse effects, as described in later clinical trials, appear to be associated with patient characteristics such as age, gender, body weight, and dosage [17,43]. A recent meta-analysis discovered that individuals with inflammatory bowel disease or other immune-mediated disorders treated with JAK inhibitors have a considerably higher risk of herpes zoster infection (RR 1.57, 95% CI 1.04–2.37) [44]. However, the particular impacts and mechanisms via which filgotinib may influence these AEs are unknown, needing additional clinical investigation. In-depth examination of filgotinib-related adverse events has revealed new signs, such as atrial fibrillation, alopecia, elevated blood creatinine levels, pulmonary embolism, epididymitis, and respiratory failure. While some of these incidents are indicated on the drug’s label, others have yet to be included. Westhovens et al. identified temporary increases in blood creatinine levels and moderate elevations in liver enzymes in filgotinib-treated RA patients, but no indication of drug-induced hepatocellular impairment [45]. Genovese et al. conducted a safety analysis of seven clinical trials, including FINCH 1–4 and DARWIN 1–3, discovered that the annual treatment rates for major adverse cardiovascular events (MACE) in RA patients receiving filgotinib at 100 mg and 200 mg doses were 0.6 per 100 patient-years (PY) and 0.3 per 100 PY. Over 52 weeks, the most common occurrence was venous thrombosis, which occurred at rates comparable to adalimumab (0.3/100 PY) and methotrexate (0.5/100 PY) [46]. All MACE cases occurred in patients who had at least one cardiovascular risk factor. Furthermore, three individuals on 200 mg filgotinib developed gastrointestinal perforation, with NSAID and corticosteroid use being relevant factors [47]. A phase III trial additionally discovered that RA patients treated with filgotinib had greater mean creatine kinase, low-density lipoprotein, and high-density lipoprotein cholesterol levels than placebo [16]. Dyslipidemia, a major risk factor for cardiovascular and cerebrovascular disorders, should be closely monitored throughout filgotinib treatment. While drug-induced cardiac events are relatively rare, this research suggests a disproportionate reporting of cardiac and respiratory events in association with filgotinib. In order to minimize these potential signals, medical professionals should screen patients for pulmonary and cardiac symptoms before starting filgotinib medication, taking these possible correlations into account during clinical assessments.

Additionally, filgotinib has been associated with signals suggesting potential impacts on the male reproductive system, according to studies by Reinisch and Hellstrom et al [48,49]. Filgotinib was potential found to be teratogenic and embryolethal in rats and rabbits in investigations pertaining to embryonic and fetal development. At an equal dose of 200 mg, the same effect was noted in humans. Filgotinib has been associated with signals suggesting potential impacts on male fertility, warranting further investigation. In order to determine whether there are any additional significant safety issues, the current MANTA research is designed to evaluate the testicular safety of filgotinib in adult males with moderately to severely active inflammatory bowel disease [50]. Although some differences in ROR values were observed across sex and age subgroups, no statistically significant interactions were detected. This finding suggests that the observed patterns likely reflect real-world reporting distributions rather than definitive biological differences. Further validation in larger cohorts and prospective studies is warranted to substantiate these observations.

This is significant because, like other JAK inhibitors, filgotinib has been given a black box warning by the FDA because it has been associated with signals suggesting an increased reporting of major adverse cardiovascular events, thrombosis, mortality, serious infections, and cancers. The findings from trials using tofacitinib served as the main basis for this warning, which was later expanded to include all JAK inhibitors. The safety profile of filgotinib was further supported by the fact that most of the safety signals found in the FAERS database were very similar to adverse events previously seen in clinical trials of the drug. The FAERS data also showed gastrointestinal complications, such as colonic erosions and ileal ulcers, which echo the higher risk of gastrointestinal perforation reported in the SELECTION trial when filgotinib was used simultaneously with NSAIDs or corticosteroids. Laboratory abnormalities similar to those seen in this study, including higher blood creatinine, moderate transaminase elevations, and dyslipidemia, were also observed by the FINCH studies. The significance of ongoing pharmacovigilance for filgotinib across a range of demographics and treatment contexts is highlighted by these consistent findings, which also support the validity of our findings. The positive effectiveness and safety results from the SELECTION and FINCH programs in other immune-mediated disorders indirectly support the prospective use of filgotinib in AS, despite the current dearth of large-scale phase III trials that specifically target AS.

There are various limitations on this study. Firstly, the limited clinical use of filgotinib and its recent regulatory approval have resulted in inconsistencies in reporting quality. Signal detection was conducted at the PT level, where near-synonymous or clinically overlapping PTs may exist. This overlap could potentially inflate signal strength associated with disease indications. Therefore, caution is warranted when interpreting the detected signals. Secondly, whereas earlier studies have indicated age as a significant predictor of adverse events due to JAK inhibitors, the tiny number of reports including older patients in this study may restrict the accuracy of subgroup analysis. Thirdly, systematic bias may also be introduced by regional differences in reporting. The findings’ external validity and generalizability may be constrained by this imbalance. Lastly, even though this study used ROR, PRR, BCPNN, and MGPS approaches, the FAERS database only captures immediate post-marketing observations. It does not have systematic follow-up or the capacity to account for any confounding variables. These limitations make it impossible to draw firm conclusions about causative linkages and impede a thorough evaluation of filgotinib’s long-term safety profile. Accounting for the pathophysiological variability among different diseases is crucial to clarifying the underlying processes of these results, particularly if symptoms associated with the underlying disease appear. In future studies the implementation of a standardized HLT/SMQ aggregation strategy, combined with the inclusion of positive and negative control events, will be employed to further evaluate and strengthen the expected performance and stability of the analytical workflow.

## 5. Conclusions

A thorough examination using actual data from the FAERS database revealed 17 SOCs that were impacted by filgotinib’s ADE, primarily in the areas of heart, respiratory, and digestive disorders. The findings in the medicine package insert were in accordance with common ADEs as pneumonia, herpes zoster, dyslipidemia, and urinary tract infections. Simultaneously, we noted notable hyperintense results in muscles and connective tissue diseases such periostitis and oligoarthritis, as well as functional disorders of the gastrointestinal tract like erosion of the large intestine and blockage of mesenteric arteries. Atrial fibrillation, baldness, increased blood creatinine, pulmonary embolism, respiratory failure, and osteopenia were among the other novel ADEs that were discovered.

## Supporting information

S1 Table The invalid PT signal detection results of ADEs related to filgotinib. (XLSX)

S2 Table Positive signals of filgotinib adverse events in males. (XLSX)

S3 Table Positive signals of filgotinib adverse events in females. (XLSX)

S4 Table Positive signals of filgotinib adverse events in the 18–65 age group. (XLSX)

S5 Table Positive signals of filgotinib adverse events in the age group above 65. (XLSX)

S6 Table Basic information of filgotinib-related ADE reports after sensitivity analysis. (XLSX)

S7 Table Positive signal adverse events of filgotinib filed by physicians at the PT level from FAERS data. (XLSX)
